# Comparison of orthodontic clear aligners and fixed appliances for anterior teeth retraction using finite element analysis

**DOI:** 10.6026/9732063002001187

**Published:** 2024-09-30

**Authors:** Chethan Thimmaiah, Geetika Tomer, Raghu Devanna, Aseem Sharma, Tanushree Sharma, Antara Majumdar, Abhaya Chandra Das

**Affiliations:** 1 MDS, Orthodontist, Private Practitioner, #1085, 2nd Main road, Vivekanada nagar, Mysore-23, Karnataka, India; 2 Professor, Department of Orthodontics and Dentofacial Orthopedics, Faculty of Dental Sciences, SGT University, Gurugram, Haryana, India; 3 Reader, Department of Orthodontics, KIMS Dental College and Hospital, KIMS Research Foundation, Amalapuram-533201, East Godavari District, Andra Pradesh, India; 4 Reader/Associate Professor, Department of Orthodontics and Dentofacial Orthopedics, HIDS, Paonta Sahib, Himachal Pradesh, India; 5 Assistant Professor, Department of Dentistry, Shri Mata Vaishno Devi Institute of Medical Excellence (Medical College & Associated Hospital, SMVDNSH), Kakryal, Jammu and Kashmir, India; 6 Intern, Kalinga Institute of Dental Sciences, KIIT University, Bhubaneswar, Odisha, India; 7 Department of Periodontics and Oral Implantology, Institute of Dental Sciences, Siksha 'O' Anusandhan (Deemed to be University), Khandagiri Square, Bhubaneswar - 751030 Odisha, India

**Keywords:** Anterior teeth, clear aligner, appliance, enhanced structure, finite element analysis, torque

## Abstract

Orthodontic clear aligners are utilised in order to correct mal-positioned teeth. The comparative effectiveness of transparent aligners and fixed appliance therapy using finite analysis has not been thoroughly studied. Therefore, it is of interest to evaluate the superiority of clear aligners over fixed orthodontic therapy in anterior tooth retraction using finite element analysis (FEA). A maxillary dentition lacking 1stpremolars and with periodontal ligaments was represented by 3D digital models. A transparent aligner, brackets and archwire were produced in a 3D printed lingual retractor. Five distinct models of clear aligners for maxillary anterior internal retraction were designed and created as part of the study: Model M0-Control, Model M1-Posterior Micro-implant, Model M2-Anterior Micro-implant, Model M3-Palatal Plate, Model M4-Enhanced structure and Model A0- Fixed Appliance. The study result has shown that the crown-root of the central incisor's sagittal displacement varied least in the improved clear aligner Model M 3. Nonetheless, noticeable differences in the patterns of tooth movement were noted between the fixed appliance model and the clear aligner models. The teeth's movement pattern stayed the same in the clear aligner models. Compared to transparent aligner types, the fixed appliance shown greater anterior torque control and improved safety for the posterior anchorage teeth. It has been demonstrated that clear aligners are non-invasive, aesthetically acceptable and effective treatment modality.

## Background:

Teeth malocclusion can have an impact on a person's social behaviour and facial appearance. Several types of fixed appliances have been used in various treatment techniques to correct malocclusion. Due to several drawbacks of fixed appliances such as; unesthetic look, difficulty in cleaning, clear aligners were created as a patient's friendly appliance. In regards of comfort and appearance, clear aligners provide better treatment experiences than traditional braces [1]. Clear aligner therapy (CAT) was first offered by Align Technology (Align Technology, California, USA) in 1997. Based on computer-simulated tooth movement stages, serial thermoplastic aligners were created in this approach. In order to gradually realign mismatched teeth to their intended positions, each aligner is worn for one to two weeks [2]. Earlier clear aligners were thought to be limited to treat only mild to moderate anterior crowding and other straightforward orthodontic issues. As computer technology has advanced and the biomechanical characteristics of aligner materials have gradually come to light, CAT has proven to be capable of handling increasingly complicated cases, including those that need for tooth extraction. Because clear aligners are not adequately strong to hold their initial shape, torque loss may occur. Because the incisors are meant to be retracted, a certain amount of incursion is purposefully added during setup [3, 4]. Numerous parameters, including aligner material, trimming design, edge extension, use of attachments, step increment and length and thickness, might impact the precision of orthodontic aligner therapy [5].

For protrusion situations, fixed orthodontic maxillary micro-implant anchoring systems offer a safe and effective treatment option [6]. On the other hand, utilising a combination of power ridges, mini-screws, overtreatment or power arms is necessary to optimise anterior torque control and guaranteed posterior anchorage during anterior retraction in order to provide accurate control over the three-dimensional movement of teeth using transparent aligners. Nevertheless, there are currently a number of drawbacks to both clear aligners and fixed orthotics, such as the possibility of micro-implant-related damage, aesthetic problems and an increase in unwanted reciprocating motion [7, 8]. Xia *et al.* have created two innovative design models for clear aligner retraction in order to maximise efficiency, reduce invasiveness and improve aesthetic appeal while retracting anterior teeth during clear aligner therapy. A lingual retractor and a transparent aligner in the form of a palatal plate are used in the modification [9]. Finite element analysis was used to assess 3D printing technique for superior anterior tooth anchoring. It is anticipated that the use of clear aligners in conjunction with tongue retractors will improve the practicality and effectiveness of anterior tooth retraction [9]. Light-cured shape memory resins or conventional thermoplastic materials can be used to create orthodontic clear aligners. It is crucial to carefully plan the shape of the aligner and its composite force system structure [10]. To achieve anchoring and to prevent anterior teeth retraction, several adjustments to the clear aligner are advised such as micro implant, enhanced structures. To efficiently regulate the torque of the maxillary anterior teeth, it was suggested that the clear aligner design incorporate power ridges. For the retraction of anterior teeth, micro-implant anchoring composite force devices has been investigated [9, 11].

Finite analysis (FE) analysis is a non-invasive technique that uses PDL reflections to mimic orthodontic treatment and forecast phenomena that are challenging to study by in vitro method. A sophisticated method for modelling the mechanical changes that occur during orthodontic treatment is three-dimensional finite element analysis, which has an emphasis on load design, material qualities, contact interactions and structural model layout [1]. After force loading, the initial tooth movement can be computed instantaneously using finite element analysis. Analysis of the stress and strain response to external pressures in residential structures has been done extensively using it in biomechanics [12]. There are limited studies on biomechanical analyses related to the clinical effectiveness of retractions of anterior teeth compared to fixed appliances. Therefore, it is of interest to assess the efficacy of clear aligner over fixed orthodontic treatment in anterior tooth r etraction using finite element analysis.

## Materials and Methods:

The present in vitro study was done in the department of Orthodontics after obtaining the ethical clearance and consent from the participants. A patient was chosen from the Department of Orthodontics who had a permanent dentition and a protrusion of maxillary bone that required the extraction of the first premolar. Cone-beam computed tomography evaluation was done for all the participants. The study's inclusion criteria were full jaw development and the presence of all teeth, with the exception of third molars.

## 3D model construction:

A maxillary dentition devoid of periodontal ligaments and first premolars was shown in three-dimensional digital models. 5 design models for clear aligner maxillary anterior internal retraction were created as part of the project; i) Model M0-Control, ii) Model M1-Posterior Micro-implant, iii) Model M2-Anterior Micro-implant, iv) Model M 3 -Palatal Plate, v) Model M4- Enhanced structure, and vi) Model A0- for Fixed Appliance. Enhanced structure was done with partially thickened CA. The modification of clear aligner was done in according to Xia et al study [9]. By applying an external offset with a thickness of 0.80 mm to the post-retraction model, the clear aligner was created. In one of the clear aligner retraction models, a 3D printed palatal plate and lingual retraction hook were paired with a clear aligner. The anterior teeth were regarded as a retraction unit in this simulation. Through the base plate, the palatal plate and lingual retractor were connected to the tooth surface. For regulated tooth movement, the centre of resistance (CR) is thought to be the essential reference point. The retraction unit's centre of resistance (CR) was used to calculate the height of the linear retraction hook. The retraction unit models were allocated the characters of rigidness. The posterior teeth's alveolar ridge roof served as the exact location of the force application point (level 0).

For FE analysis, ANSYS Workbench 14.0 (Ansys, Pennsylvania, USA) imported all of the components. For calculations, a 3D printed lingual retractor with brackets, clear aligner and archwire was made. The term "crown-root differential displacement" refers to the diversity among the root tip and crown displacements. The point where the discrepancy dislocations of the anchorage units are almost equal to zero is known as the centre of resistance (CR) level. It was assumed that every research subject had isotropy, continuous homogeneity, and a constitutive model of linear elastic material. Using the C3D10M element type, the three-dimensional models were meshes. The global coordinate system's Y-axis indicates the vertical direction; positive values are defined as those that are perpendicular to the occlusal plane and point in the direction of the root. The distal direction is represented by the X-value, which has positive values allocated to it. The X-axis denotes the mesiodistal direction. The bucco-palatal direction is represented by the Z-axis. The cusp tip and root apex of the canines, as well as the incisal midpoint and root apex of the incisors, were chosen as reference points. Software was used to do nonlinear iterative computations, which produced thorough data including the displacement of tooth and aligners as well as the von-Mises equivalent stress that the PDL and aligners endured.

## Results:

Table 1 lists the characteristics of the materials and their nods for various buildings. The improved clear aligner Model M 3 demonstrated least amount of variation in the sagittal displacement of the crown-root of the central incisor, as indicated in Table 2. Nonetheless, noticeable differences in the patterns of tooth movement were noted between the fixed appliance model and the clear aligner models. The teeth's movement pattern stayed the same in the clear aligner models. Compared to transparent aligner types, the permanent appliance gives greater anterior torque control and improved protection for the posterior anchorage teeth. The canine, lateral incisor and central incisor all showed positive crown-root displacement differences at level 4. At level 5, the corresponding crown-root displacement caused these numbers to turn negative. Under the loading situations of all 5 clear aligner models, it was discovered that the sagittal movement outlines of the canine, lateral incisor, and central incisor were comparable. The central incisor's root and crown, however, showed buccal movement in the fixed appliance model. The lingual incisor's root shifted lingually, but the crown moved buccally. Table 2 shows that, for the central incisor, Model M3 had the least crown-root displacement variation. The lateral incisors and canines of Model M4 (enhanced structure) showed the least variations. Model M3 showed the least amount of crown movement for the central incisor in Table 3 out of all the clear aligner models. In a similar vein, the lateral incisor displacements for Model M4 were the least. In all five of the clear aligner models, the stress distribution and magnitude on PDL were comparable. The displacement of the premolar crown under various loading scenarios in both sagittal and vertical directions is shown in Table 4 and Table 5. Fixed appliance showed largest crown displacement under sagittal load and largest displacement with clear aligner with Model M2 under vertical load for premolar.

## Discussion:

Clear aligners are becoming more and more popular as a treatment for misaligned teeth, but there are still a number of questions about how well the system manages tooth movement. Numerous studies on the biomechanics of potential tooth movement using clear aligners have been conducted. One benefit of 3D FE analysis is that it can compute stress and accounts for periodontal tissues [2]. In this study; we examined the biomechanical variations between multiple invisible orthodontic devices during anterior retraction and used numerical simulations to assess the anterior retraction process in various orthodontic designs. The fixed appliance retraction model and the clear aligner retraction model were evaluated. It was discovered that the tendency of tooth movement in clear aligner models remained unchanged by minor biomechanics differences between the various models and by the installation of force systems. Compared to the other four clear aligners, the Model M3 showed better torque control and offered more protection for posterior anchorage teeth. Both the fixed appliance and the transparent aligner displayed unique biomechanical characteristics. Lingual tilting and extrusion in the anterior teeth were consistently seen in the clear aligner models [13]. The study conducted by Liu *et al.* showcased the effectiveness of anterior mini-screws and elastics in accomplishing incisor intrusion and palatal root torqueing [14]. For clear aligner therapy, Jiang *et al.* assessed tooth behaviours under various maxillary incisor retraction methods. They came to the conclusion that lingual root movement during incisor retraction was caused by the incorporation of intrusion displacement in clear aligners [2]. Comparing Model M2 to Models M0 and M1, the current investigation found that Model M2 had better torque and vertical control over the anterior teeth. We discovered that the most accurate torque and vertical control was displayed by the Model M3. This is because of the palatal plate's involvement in uniting with the posterior teeth to produce a stronger anchorage unit, as well as its stabilising and cushioning effect during the retraction process. Even with the clear aligner, the maximum von-mises stress was still much lower than the stationary appliance's.

Compared to the clear aligners, the fixed appliance model's teeth showed a much different initial displacement tendency. The lateral incisor was most noticeably affected by the fixed appliance [9]. In comparison to the transparent aligner models, the fixed appliance model's tooth displacement magnitude was noticeably smaller. This was in line with earlier research conducted by Ke *et al.* and Robertson *et al.* [15, 16]. Jin *et al.* concluded that enhanced structure in clear aligners is helpful and it allows force delivery in compliance with optimal principles of biomechanics during the extraction space closure [17]. It is association with our findings. According to Baldwin *et al.* clear aligners are not as rigid as fixed appliances when it comes to preventing tipping [18]. Wang *et al.* came to the conclusion that while aligners move a little more slowly than fixed appliances, their periodontal health is improved [19]. The movement trend and the stress distribution in PDL were similar for all 5 of the clear aligner models. Nonetheless, Tang *et al.* came at the conclusion that the fixed appliance model's PDL stress was significantly lower than the clear aligner models' [20]. Since it is practically impossible to replicate the exact same biological substance in a mechanical model, more research on finite element analysis through comprehensive clinical studies is required to quantitatively validate our findings. By combining FE analysis with clinical investigations for mutual validation, this study's significance will be increased.

## Conclusion:

The permanent appliance form offers greater anterior torque control and improved protection for the posterior anchorage teeth compared to transparent aligner types. The way the teeth moved with all five of the clear aligners was the same. The modified palatal plate structure clear aligner Model M3 was used to improve torsional control. A non-invasive, visually beautiful, comfortable and effective procedure has been demonstrated by clear aligner.

## Figures and Tables

**Table 1:** Properties and element number of various structures

**Component**	**Young's Modulus (MPa)**	**Poisson's Ratio**	**Nodes**	Elements
Teeth	18557	0.32	231643	129345
Periodontal ligament	0.57	0.46	121016	61365
Alveolar bone	138 00	0.31	208765	119753
Cortical bone	13600	0.32	206854	118574
Clear aligner	80848	0.32	121244-158131	62200-6686
3D printed	228000	0.32	50142	22646
Archwire	200100	0.31	23456	10236
Micro implant	217000	0.31	29164	13245
Bracket	112000	0.31	4674	2506

**Table 2:** Displacement of the maxillary anterior teeth's crown and root under various loading scenarios in the sagittal direction (mm)

**Component**	**Young's Modulus (MPa)**	**Poisson's Ratio**	**Nodes**	**Elements**
Teeth	18557	0.32	231643	129345
Periodontal ligament	0.57	0.46	121016	61365
Alveolar bone	138 00	0.31	208765	119753
Cortical bone	13600	0.32	206854	118574
Clear aligner	80848	0.32	121244-158131	62200-6686
3D printed	228000	0.32	50142	22646
Archwire	200100	0.31	23456	10236
Micro implant	217000	0.31	29164	13245
Bracket	112000	0.31	4674	2506

**Table 3:** Displacement of the maxillary anterior teeth's crown and root under various loading scenarios in the vertical direction (mm)

**Model**	**Central incisor**		**Lateral incisor**		**Canine**	
	Crown	Root	Crown	Root	Crown	Root
Model M0	-3.88E-02	1.12E-02	-4.37E-02	1.27E-02	-2.79E-02	2.12E-02
Model M1	-3.16E-02	1.14E-02	-4.58E-02	1.36E-02	-2.82E-02	2.32E-02
Model M2	-3.45E-02	1.08E-02	-4.02E-02	1.38E-02	-2.88E-02	2.26E-02
Model M3	-3.02E-0.2	8.76E-03	-3.85E-0.2	1.12E-03	-2.67E-0.2	2.02E-03
Model M4	-4.09E-02	1.01E-02	-3.62E-02	1.36E-02	-2.31E-02	2.17E-02
Model A0	4.87E-05	6.93E-04	8.65E-05	7.24E-04	-4.58E-04	-1.18E-04

**Table 4:** Crown displacement in a sagittal direction (mm) of the maxillary posterior teeth under various loading models

**Model**	**2nd premolar crown**	**1st molar crown**	**2nd molar crown**
Model M0	-3.41E-02	-2.14E-02	-2.08E-03
Model M1	-3.42E-02	-2.36E-02	-2.13E-03
Model M2	-3.51E-02	-2.32E-02	-2.21E-03
Model M3	-3.62E-03	-2.12E-02	-1.63E-03
Model M4	-3.16E-04	-2.16E-02	-1.62E-03
Model A0	6.15E-03	1.01E-04	1.56E-04

**Table 5:** Displacement of the maxillary posterior teeth's crowns in a vertical direction (mm) under various loading models

**Model**	**2nd premolar crown**	**1st molar crown**	**2nd molar crown**
Model M0	7.46E-03	8.06E-03	-2.07E-03
Model M1	8.46E-03	5.24E-03	-2.12E-03
Model M2	8.53E-03	5.75E-03	-2.25E-03
Model M3	6.82E-03	3.46E-03	-1.43E-03
Model M4	1.08E-04	-5.36E-04	-2.52E-04
Model A0	1.17E-04	-4.87E-03	-5.17E-04
